# Localization of MEG human brain responses to retinotopic visual stimuli with contrasting source reconstruction approaches

**DOI:** 10.3389/fnins.2014.00127

**Published:** 2014-05-27

**Authors:** Nela Cicmil, Holly Bridge, Andrew J. Parker, Mark W. Woolrich, Kristine Krug

**Affiliations:** ^1^Department of Physiology, Anatomy and Genetics, University of OxfordOxford, UK; ^2^Nuffield Department of Clinical Neuroscience, FMRIB Centre, John Radcliffe Hospital, University of OxfordOxford, UK; ^3^Department of Psychiatry, Oxford Centre for Human Brain Activity, Warneford Hospital, University of OxfordOxford, UK

**Keywords:** magnetoencephalography (MEG), brain imaging, source localization, retinotopy, vision (ocular), fMRI

## Abstract

Magnetoencephalography (MEG) allows the physiological recording of human brain activity at high temporal resolution. However, spatial localization of the source of the MEG signal is an ill-posed problem as the signal alone cannot constrain a unique solution and additional prior assumptions must be enforced. An adequate source reconstruction method for investigating the human visual system should place the sources of early visual activity in known locations in the occipital cortex. We localized sources of retinotopic MEG signals from the human brain with contrasting reconstruction approaches (minimum norm, multiple sparse priors, and beamformer) and compared these to the visual retinotopic map obtained with fMRI in the same individuals. When reconstructing brain responses to visual stimuli that differed by angular position, we found reliable localization to the appropriate retinotopic visual field quadrant by a minimum norm approach and by beamforming. Retinotopic map eccentricity in accordance with the fMRI map could not consistently be localized using an annular stimulus with any reconstruction method, but confining eccentricity stimuli to one visual field quadrant resulted in significant improvement with the minimum norm. These results inform the application of source analysis approaches for future MEG studies of the visual system, and indicate some current limits on localization accuracy of MEG signals.

## Introduction

Magnetoencephalography (MEG) measures magnetic fields emitted by neuronal electrical activity and thus allows the non-invasive recording of neuronal signals with millisecond temporal resolution (Hämäläinen et al., 1993). MEG has the potential to extend findings from electrophysiological studies in the visual systems of animals by recording neuronal activity across the whole brain in human viewers as they respond to visual stimuli. The high temporal resolution of MEG can complement results from functional MRI (fMRI), a human neuroimaging method that has good spatial resolution (approximately 1 mm) but provides an indirect measure of neuronal function with low temporal resolution relative to neuronal spiking activity (Logothetis et al., 2001; Logothetis and Wandell, 2004).

Although the magnetic fields measured by MEG pass through brain, skull and skin with minimal smearing [in contrast to the electrical potentials measured by electroencephalography (EEG)], localization of brain sources of MEG signals remains an ill-posed problem. The number of independent measurements of the signal is on the order of a few hundred sensors, whilst the possible spatial configurations of cortical sources giving rise to that signal is several orders of magnitude greater; hence, MEG measurements alone cannot constrain a unique solution to the inverse problem of source reconstruction (Hämäläinen et al., 1993).

A current approach to overcome this limitation is to impose prior constraints on the source solution, informed by assumptions about the brain activity patterns that give rise to the MEG signal. Different approaches to source reconstruction have been developed, incorporating different prior assumptions. The minimum norm estimate constrains the source solution by requiring that absolute activity amplitudes across the brain be as small as possible on average (Dale and Sereno, 1993; Hämäläinen and Ilmoniemi, 1994). Additionally, sources can be limited to the cortical mantle and a depth-weighting parameter used to counter the implicit bias of these assumptions toward superficial, spatially spread currents (Lin et al., 2006). On the other hand, brain activity can be assumed to be sparse, i.e., occurring in discrete cortical “patches”, which in certain tasks may have a bilaterally correlated response (Pascual-Marqui et al., 1994). These sparseness and correlation parameters can be inferred from the data using Bayesian techniques, for example in the multiple sparse priors approach (Mattout et al., 2007; Friston et al., 2008; Henson et al., 2009). Related algorithms have been the basis of other source reconstruction approaches (Moradi et al., 2003; Poghosyan and Ioannides, 2007; Cottereau et al., 2011).

Alternatively, a spatial filtering algorithm known as beamforming can be employed to estimate the time-course of activity at each source location, independently of all other sources, and can be extended to evaluate signals within a frequency band of interest (van Veen et al., 1997; Robinson and Vrba, 1999; Barnes et al., 2006; Hillebrand and Barnes, 2011). Neuronal responses may oscillate at a particular frequency due to the internal properties of the processing networks involved (Wang, 2010), or a rhythmic change in the presented stimulus can evoke brain responses in a particular frequency band (Cottereau et al., 2011). In both cases, such frequency-related information can be used to focus source analysis onto a subspace of the measured MEG signal.

For visual neuroscience research, MEG source reconstruction methods should assign sources of early visual responses to occipital cortex and resolve activity arising from different occipital locations. However, with many contrasting reconstruction approaches available, it is not yet clear which prior assumptions are most appropriate for localizing MEG signals arising from the human visual system, specifically those from early cortical visual areas V1, V2, and V3.

The current gold standard for high spatial resolution of human visual brain activity is fMRI, which has been used to identify the retinotopic boundaries between visual areas, allowing comparison of responses along the visual hierarchy (Engel et al., 1994; Sereno et al., 1995; DeYoe et al., 1996; Wandell et al., 2005). Retinotopic mapping in early visual cortical areas of the human brain follows well-established patterns. In angular retinotopy, upper visual field locations are represented in ventral subregions of early visual areas, whilst lower visual field locations are represented in dorsal subregions. Left and right visual field locations are represented in the respective contralateral cortical hemispheres. For visual field eccentricity, the foveal region is represented at the occipital pole and representations of increasingly peripheral locations radiate anteriorly (Engel et al., 1994; DeYoe et al., 1996; Wandell et al., 2005). Comparison of the sources of the MEG signals of visual brain responses, as reconstructed by different reconstruction approaches, to fMRI retinotopic maps or regions of interest (ROIs) in the same individual should reveal which approaches can accurately localize signals arising from the visual system.

A number of studies that have evaluated MEG source reconstruction methods have compared the reconstruction of simulated electromagnetic data to their assumed sources (Hämäläinen and Ilmoniemi, 1994; Hauk, 2004; Lin et al., 2006; Trujillo-Barreto et al., 2008; Hillebrand and Barnes, 2011) and/or quantified goodness of reconstruction with a fitness measure such as model evidence rather than source localization accuracy (Mattout et al., 2007; Friston et al., 2008; Henson et al., 2009). A few studies have evaluated localization accuracy of one specific MEG source reconstruction method for real recorded visual responses, by comparing the source locations either to individuals' fMRI maps (Moradi et al., 2003; Poghosyan and Ioannides, 2007; Sharon et al., 2007; Cottereau et al., 2011) or to indirect indicators of retinotopic mapping, such as anatomical landmarks (Brookes et al., 2010; Perry et al., 2011).

We further this approach by reconstructing, for the first time, the sources of real recorded MEG signals from human viewers with three contrasting localization approaches and evaluating these reconstructions against fMRI retinotopic maps from the same individuals. Source localizations of responses to stimuli that differed either in angular retinotopy or eccentricity were compared to their independently established cortical locations in early visual areas V1, V2, V3, and V3A, defined for the individual participants by fMRI. We used large stimuli and assessed the accuracy of the extent of cortical activations rather than just one focal point in early visual areas. We focused on three methods included in freely available software packages: minimum norm (Minimum Norm Estimate, MGH/MIT Martinos Centre for Biomedical Imaging; Dale et al., 2000; Gramfort et al., 2014), multiple sparse priors (MSP in SPM8 software, FIL Methods Group, UCL; Litvak et al., 2011), and beamforming (adapted from SPM8 to work with Elekta Neuromag data; Woolrich et al., 2011). The beamformer was applied separately to early visual evoked responses and to ongoing oscillatory responses related to the stimulus flicker rate; minimum norm and multiple sparse priors were used to reconstruct early evoked responses only. A number of recent studies have incorporated information from fMRI retinotopic mapping to aid the localization of the MEG signal by placing spatial priors on the source solutions (Yoshioka et al., 2008; Hagler et al., 2009; Cottereau et al., 2012; Hagler and Dale, 2013). In contrast, our investigation focused on the reconstruction of sources from MEG signals alone, so the individual fMRI map provided an independent localization comparison.

Any justification for a combination of MEG and fMRI data needs to be based on a clear understanding of the contribution of each signal to the combined estimate. Our contribution here is based upon analyzing the quality of spatial localization of the MEG signal, using current standard methods.

## Materials and methods

### Participants

Eight participants (6 female, 2 male; mean age 31.4 ± 12.6 years, range 22–58 years) took part in the experiment, although not all participants completed all measurements. Further details are given later. All participants had normal, or corrected to normal, visual acuity. The participants had no neurological or psychiatric illness, no brain injury, and were not taking any medications that might affect the nervous system. The research was approved by the University of Oxford's Central University Research Ethics Committee (CUREC), in accordance with the regulatory standards of the Code of Ethics of the World Medical Association (Declaration of Helsinki). Written informed consent was obtained from all participants who were not investigators of the project.

### MEG retinotopy

#### Data collection and pre-processing

***Stimuli***. Visual stimuli were projected onto a back-projection screen in the MEG scanner in front of the participant with a Panasonic® DLP (Digital Light Processing) based projector (PT-D7700E). Refresh rate was 60 Hz (all MEG data were lowpass filtered at 40 Hz prior to source reconstruction, see below). Distance between viewers' eyes and screen was 1500 mm and projected screen size was 390 × 290 mm, corresponding to 14.8 × 11.0° of visual angle. Accurate stimulus onset times were recorded with a photodiode (sampling rate 1000 Hz) placed over a small black square (8 × 8 mm) located in the bottom-left corner of the stimulus screen; this square flashed to white for 100 ms on the first frame of each stimulus onset (the photodiode blocked this flash from being seen by the participant). Participants passively viewed stimuli whilst maintaining central fixation.

Black-and-white checkerboard quadrant stimuli were presented to 6 participants with a Cambridge Research Systems VSG 2/5 graphics generator run with a Dell laptop (Subjects 1–4), or with Presentation® (Neurobehavioral Systems, Inc.) running on a Samsung R710 laptop (Centrino 2 P7450 processor, nVIDIA GeForce 9300M graphics card) (Subjects 5 and 6). Stimulus parameters were identical in both set-ups. Each quadrant extended 0–5.4° eccentricity, presented either in the upper left (UL), upper right (UR), lower left (LL) or lower right (LR) visual field. Quadrants contained 6 checks along the radius and the arc, decreasing in size by a factor of 1/*d*, where *d* is distance to apex. A black fixation point (radius 0.25°) was present at the apex. Each stimulus was presented for 1000 ms with no inter-stimulus interval. Each block of quadrant stimuli consisted of 25 full-cycle rotations (UR, UL, LL, LR positions). 6 blocks were collected per participant.

Black-and-white checkerboard concentric ring and quarter-ring stimuli were presented with Presentation® software, as above, for all participants. Rings had 12 checks around the circumference and 3 checks along the radius, and were presented at three eccentricities: ECC 1 (0–0.75°), ECC 2 (1.0–2.0°), and ECC 3 (3.0–5.4°). These eccentricity bands were selected to activate areas of similar size across cortex according to foveal magnification ratios, and extend approximately 3 cm into the calcarine sulcus; doubling maximum ring size would have further increased this extent by approximately 1 cm only (Wandell et al., 2005; Horton, 2006). Quarter-rings were formed from rings by masking out all but either the upper right or lower right quadrant of the visual field, resulting in 6 quarter-ring stimuli (upper right: U-ECC 1, U-ECC 2, and U-ECC 3; lower right: L-ECC 1, L-ECC 2, and L-ECC 3). Ring and quarter-ring stimuli were presented for 1000 ms in a pseudo-randomized order with a variable inter-stimulus interval of 600, 800, or 1000 ms (selected pseudo-randomly). Datasets for rings were recorded for 7 participants (Subjects 1–3 and 5–8) with 4 blocks of 150 stimuli per participant. Datasets for quarter-rings were recorded for 5 participants (Subjects 1–2 and 6–8) with 5 blocks of 180 stimuli per participant.

All stimuli cycled through complete black-to-white-to-black or white-to-black-to-white contrast reversal at a rate of 4 Hz, i.e., the presented checkerboard pattern changed every 125 ms. This induces oscillatory brain responses at the second harmonic, a rate of 8 Hz. Stimuli were presented on a mid-gray background (mean luminance, 25 cd/m^2^); Michelson contrast was 99%.

***MEG scanner and data acquisition***. MEG data were collected with an Elekta Neuromag VectorView® MEG scanner at the Oxford Centre for Human Brain Activity (OHBA), Department of Psychiatry, University of Oxford, Warneford Hospital, Oxford, U.K. The scanner comprises 306 MEG-channel sensors (102 magnetometers, 204 planar gradiometers). Sensors were tuned prior to each MEG recording session to limit noise levels to approximately 2.5 fT/cm. Sensors that became very noisy during a recording block would be individually re-tuned at the next inter-block break, using the Neuromag automatized heating process or by eye, as necessary. Continuous MEG data were recorded at 1000 Hz sampling rate (0.3–330 Hz bandpass filter). Prior to data acquisition, all metal and other potential sources of electromagnetic interference were removed from participants. Quality of recording was confirmed by visual inspection of 1–2 min of MEG recording during quiet sitting prior to the start of the experiment. Electro-oculogram (EOG) and electrocardiogram (ECG) time-series were recorded simultaneously with MEG to track potential noise sources and artifacts. Four head position indicator (HPI) coils were attached to the participant's head and a Polhemus stylus and digitizer device were used to record the locations of fiducial points (right and left pre-auricular points (RPA, LPA) and nasion), the HPI coils, and between 40 and 80 extra digitizer points on the head surface. Prior to the recording of each stimulus block, head location in the scanner was measured with an automatic process that detected the coils. Continuous HPI recorded any head movements during data acquisition.

***Preprocessing and HPI correction***. Data were preprocessed with Elekta Neuromag® MaxFilter software (version 2.1, May 2009). MaxFilter software reduces noise in the data by suppressing magnetic interference coming from outside and inside the sensory array, using signal-space separation (SSS). The MaxMove sub-command was used to spatially co-register MEG recordings across blocks to the median head position for each individual. MaxMove continuous HPI movement compensation was also applied. Data were then epoched according to the onset of each visual stimulus (−500 to 1000 ms peri-onset).

***Artifact removal***. MEG channels with constant high noise levels as identified by visual inspection were rejected from further analysis. A maximum of two such channels was removed per participant and scan. Eye-related artifacts such as blinks were identified as deviations in the EOG recording trace. Epochs containing artifacts arising from the eyes or intermittent sensor noise were removed from further analysis. Peak-to-peak threshold for removal of eye blinks and overt eye movements was within the range 100–200 × 10^−6^ V. Maximum noise level threshold for magnetometer and gradiometer activity was within range 2–3 × 10^−12^ T and 1.5–2 × 10^−10^ T/m, respectively. In both cases, the specific threshold depended on the artifact amplitudes recorded for each individual. After artifact removal, in all cases there remained at least 95 trials per stimulus per participant.

#### Source reconstruction of MEG signals

Brain sources of MEG signals were localized using three different reconstruction approaches. The following sections detail the source space configurations, reconstruction approaches, and statistical methods used. Table 1 provides a summary of these details along with the resultant localization accuracies for responses to quadrant stimuli.

**Table 1:** **Source reconstruction method details for all localization accuracy comparisons**.

**Software package**	**MEG signal**	**Source space configuration (vertex spacing)**	**HPI-MRI co-registration algorithm**	**MEG reconstruction algorithm**	**Time window**	**Statistical comparison**	**Mean V1, V2, and V3 localization accuracy % (quadrants)**
MNE	Early evoked response	Individual's cortical surface mesh (3.1–4.9 mm)	Iterative closest point (ICP)	MNE	20 ms, centered on FRP	dSPM F statistic, 500 ms baseline (previous stimulus)	77.9 (mean *SD*: 24.7)
OSL	Early evoked response	Brain volume (4 mm)	Iterative closest point (ICP)	Beamformer (1–40 Hz)	20 ms, centered on FRP	*t*-test of trial-wise difference between stimuli	69.1 (mean *SD*: 36.5)
OSL	Stimulus frequency tag (8 Hz)	Brain volume (4 mm)	Iterative closest point (ICP)	Beamformer (7–9 Hz)	200–1000 ms post stimulus onset	*t*-test of trial-wise difference between stimuli	66.0 (mean *SD*: 39.3)
SPM8	Early evoked response	Inverse-normalized cortical surface mesh (4.9 mm)	Iterative closest point (ICP)	MSP	40 ms^*^, centered on FRP	*t*-test of trial-wise difference between stimuli	54.9 (mean *SD*: 33.8)
SPM8	Early evoked response	Inverse-normalized cortical surface mesh (4.9 mm)	Iterative closest point (ICP)	IID	40 ms^*^, centered on FRP	*t*-test of trial-wise difference between stimuli	57.3 (mean *SD*: 28.5)
SPM8	Early evoked response	Inverse-normalized cortical surface mesh (4.9 mm)	Iterative closest point (ICP)	MSP	50–200^*^ ms post stimulus onset	*t*-test of trial-wise difference between stimuli	36.9 (mean *SD*: 25.6)
SPM8	Early evoked response	Inverse-normalized cortical surface mesh (4.9 mm)	Iterative closest point (ICP)	IID	50–200^*^ ms post stimulus onset	*t*-test of trial-wise difference between stimuli	54.8 (mean *SD*: 38.5)

* *Gaussian-weighted average over the time period*.

***Anatomical MRI data collection***. Anatomical magnetic resonance imaging (aMRI) data were collected with a 3.0 Tesla TIM Trio scanner, located at the University of Oxford Centre for Clinical Magnetic Resonance Research (OCMR). One T1 scan was taken for each participant using a standard structural magnetization-prepared rapid gradient echo (MPRAGE) sequence (130 Hz/pixel, flip angle = 8°, TR/TE/TI = 2040 ms/4.7 ms/900 ms). Orientation of scan acquisition was transverse (192 × 1 mm slices) with an inplane resolution of 1 × 1 mm.

***Source space modeling and HPI-MRI alignment***. Individuals' anatomical surfaces, to which MEG data were co-registered, were created from the aMRI data with Freesurfer software *recon-all* process (default parameters) (http://surfer.nmr.mgh.harvard.edu; Dale et al., 1999; Fischl et al., 1999). Correct segmentation of white/gray matter for cortical surfaces was confirmed by eye. FreeSurfer's *watershed* algorithm was used to reconstruct the inner skull, outer skull and outer skin surfaces from the individuals' aMRI data and to estimate the boundary element model (BEM) compartments. BEM compartments are used to specify the model for the electrical conductivity geometry of the head. A “single shell” forward model based upon this BEM was used in all source reconstruction methods.

Minimum norm reconstructions were implemented with MNE software (see Minimum norm estimate (MNE) reconstruction), which creates each individual's source space based upon each individual's cortical surface. Individuals' source spaces contained 10242 sources per hemisphere (corresponding to 3.1 mm source spacing) for all participants except Subjects 2, 3, and 4, for whom the anatomical scan and cortical surface reconstructions permitted a maximum of 4098 sources per hemisphere (corresponding to 4.9 mm source spacing). The specific resolution for each individual was limited by the *mne_setup_source_space* command, which constructs the triangulated dipole grid from the reconstructed white matter surface, in the MNE analysis pipeline. Source reconstruction with multiple sparse priors assumptions was implemented with SPM8 software (see Multiple sparse priors (MSP) reconstruction). This software constructs the cortical surface meshes for the source space by inverse normalization of the canonical mesh derived from the MNI152 template brain (Mattout et al., 2007; Henson et al., 2009). These source spaces contain 4098 sources per hemisphere (corresponding to source spacing of approximately 4.9 mm (as advised by SPM8 Manual, Section 14.3, Source space modeling, p. 121). Beamformer source reconstruction did not confine activity to the cortical mesh but estimated it within the cranial volume. A source spacing of 4 mm was selected to lie reasonably within the range of resolutions utilized within the other reconstruction approaches. Table 1 lists the source space used for each reconstruction approach.

Digitized fiducial points, HPI coils and remaining digitizer points were used to align the coordinate frame of the MEG data and the structural MRI data. Locations of fiducial points were first specified on the aMRI volume and an automatic alignment procedure, using an iterative closest point algorithm (ICP), non-linearly converged the frames to optimal alignment. The beamformer utilized the same co-registration as created in the SPM8 software for the multiple sparse priors method. Co-registration for the minimum norm reconstruction was run in the MNE software package using identical positional information and equivalent ICP alignment.

***First response peak (FRP)***. The time window for all source reconstructions of the early evoked response was centered on 83 ms, representing the ascension of the FRP, which was qualitatively determined by eye. This FRP was used for all participants except for Subject 7, for whom 93 ms was used, as evoked responses for this participant were 10 ms slower to rise.

***Minimum norm estimate (MNE) reconstruction***. Data were analyzed with MNE software (Minimum Norm Estimate, MGH/MIT Martinos Centre for BioMedical Imaging; Hämäläinen and Ilmoniemi, 1994; Dale et al., 2000; Gramfort et al., 2014), time-locked to stimulus onset and averaged. A noise covariance matrix (NCM) was calculated from -500 to 0 ms prior to each stimulus onset; for quadrant stimuli, this necessarily comprised the final 500 ms of the previous stimulus presentation. Source reconstructions were performed on data bandpass filtered 1–40 Hz for 0–1000 ms post-stimulus, combining magnetometer and gradiometer measurements. Anatomically constrained dynamic statistical parametric mapping (dSPM) inverse solutions (based upon F-statistics calculated using baseline variance estimates) were generated at each cortical vertex (Dale et al., 2000). These dSPM source estimates were averaged across a 20 ms time window, centered on the FRP.

***Beamformer (early evoked response)***. Data were analyzed with an LCMV (linearly constrained minimum variance) beamformer (adapted from SPM8 to work with SSS MaxFiltered Elekta Neuromag data; Woolrich et al., 2011), using lead fields calculated from the SPM8 neuroimaging analysis package (FIL Methods Group, UCL; Friston et al., 2008; Litvak et al., 2011). The beamformer data covariance matrix and weights were averaged over all trials, and used to produce separate reconstructed sources for each trial. These were then combined in a trial-wise General Linear Model to produce a t-statistic for each source location. For quadrant stimuli, the t-statistic described the trial-wise difference between responses to a particular quadrant compared to the other quadrants, as no inter-stimulus interval baseline was available. For rings and quarter-rings, the t-statistic described the difference between responses to the stimulus vs. average baseline activity −250 to 0 ms prior to stimulus onset. Sources were reconstructed for 0–1000 ms post-stimulus, bandpass filtered at 1–40 Hz, combining magnetometers and gradiometers. Resultant t-statistic images were averaged across a time window of 20 ms, centered on the FRP.

***Beamformer (time-frequency)***. Time-frequency decomposition source analysis was performed within the 7–9 Hz frequency band, centered on 8 Hz, i.e., the 2nd harmonic of the stimulus contrast-reversal frequency. The 2nd harmonic is used because each contrast reversal of the stimulus involves two contrast changes (from black to white then white to black) and visual brain areas respond to each such change (Campbell and Kulikowski, 1972; Cottereau et al., 2011). A pilot frequency decomposition analysis on sensor activity confirmed this band contained the greatest power. A time window of 200–1000 ms was selected for source reconstruction to avoid the FRP yet utilize maximum available data for reconstruction. Resultant t-statistic images were averaged across the time window. All other parameters were identical to the initial evoked response beamformer analysis above.

***Multiple sparse priors (MSP) reconstruction***. Data were analyzed with the MSP analysis algorithm available in the SPM8 M/EEG analysis package (FIL Methods Group, UCL; Friston et al., 2008; Litvak et al., 2011). MSP contains bilaterally symmetrical a priori assumptions based upon functional anatomy, which are selected or deselected by the reconstruction algorithm according to the presence or absence of bilateral correlation components in the data (Friston et al., 2008). Sources were reconstructed separately for each trial and a t-statistic was calculated across trials to indicate significance of source activity, as for the beamformer. Time window of source reconstruction was 40 ms wide, centered on the FRP, combining magnetometers and gradiometers. Source activity results were averaged over this time window, weighted by a Gaussian centered on the FRP.

The SPM8 analysis package was also used to run reconstructions with the IID (independently and identically distributed priors) reconstruction option, which corresponds to the minimum norm approach but does not incorporate the same depth weighting and anatomical constraints as the MNE software. All other factors were identical between IID and MSP reconstructions. Since the SPM8 source reconstruction procedure reconstructs variance around the mean signal, MSP and IID reconstructions were also run using a 150 ms time window (50–200 ms post-stimulus), to encompass a greater amount of the response to stimulus onset. This wider time window did not result in improved source localization accuracy (Table 1). Therefore the shorter time window was used for the main comparisons in the present study.

***Morphing 3D source images to the individual's cortical surface***. The beamformer and MSP methods output source reconstructions in MNI152 volumetric standard space. These were converted to individuals' cortical surface format (Freesurfer) to enable comparison with individuals' fMRI retinotopic maps. The *flirt* command from FSL (FMRIB, Oxford; Jenkinson et al., 2012) generated a transformation matrix from MNI152 volumetric space to Freesurfer volumetric space and then transformed the 3D source images to Freesurfer space. Freesurfer volume images were then converted to cortical surface format *mri_vol2surf* command (Freesurfer). These surface files were then morphed onto the cortical anatomy of the individual participant with *mri_surf2surf* (Freesurfer). The correspondence between the volumetric standard space results and the native space output of the morphing procedure was carefully checked and confirmed by eye at every stage for each individual subject.

### Functional MRI retinotopy

#### Stimuli

Retinotopic quadrant and ring stimuli used for fMRI data collection were presented with the Cambridge Research Systems VSG 2/5 graphics generator with a Dell laptop. Visual stimulus parameters were identical to those used for MEG unless otherwise stated below. The quadrant stimulus rotated through 30° every TR (4000 ms) to producing traveling wave brain signals necessary for analysis with standard fMRI retinotopy software (Wandell, 1999). Similarly, concentric rings expanded every TR (4000 ms), taking 8 steps to cover the visual field 0–11.5°. Hence, although the timing of visual stimulus presentation differed between fMRI and MEG data acquisitions, identical spatial points of the stimuli in the two cases could be selected, enabling direct comparisons between the brain source locations.

#### fMRI data acquisition

Retinotopic fMRI data were acquired according to standard methods with a 3T Tesla whole-body Siemens TIM Trio scanner and a 12-channel receive-only head coil, located at the University of Oxford Centre for Clinical Magnetic Resonance Research (OCMR). EPI sequence parameters were: TE = 30 ms; TR = 4000 ms; 40 2-mm slices; 2 × 2 mm in-plane resolution; matrix = 64 × 64. For angular mapping, each run consisted of 6 cycles through 12 angular locations, corresponding to 72 volumes acquired continuously (288 s); 4 runs were collected. For eccentricity mapping, each run consisted of 6 cycles through 8 eccentricities, corresponding to 48 volumes (192 s); 3–4 runs were collected. A reduced (40 2-mm slices) T1-weighted image (3D FLASH) was also included in each functional session, acquired coronally at an in-plane resolution of 1 × 1 mm. These slices were in the same planes as the retinotopic functional images, and were used to register functional retinotopy data to the whole brain structural MRI.

#### fMRI retinotopy mapping

The fMRI retinotopic maps were generated for individual participants according to standard procedures, using either mrTools software (HeegerLab; http://www.cns.nyu.edu/heegerlab/wiki/) or mrVista software (Stanford; http://white.stanford.edu/software). Retinotopic BOLD activity maps were displayed on flat renderings of the occipito-temporal-parietal region, allowing borders between visual areas to be identified and traced. For angular retinotopy, dorsal (lower visual field) and ventral (upper visual field) subregions were defined on the left and right hemisphere for areas V1, V2, and V3. Area V3A was also defined on each hemisphere. For eccentricity, regions of interest (ROIs) representing the eccentricity bands for ECC 1, ECC 2, and ECC 3 stimuli were delineated across areas V1, V2, and V3. This fMRI retinotopic mapping procedure and combination of parameters have been used to map retinotopic visual areas across a significant number of individual subjects (Bridge and Parker, 2007; Minini et al., 2010). The definitions of areas V1-V3A according to this procedure are reliable insofar as—when combined with additional subjects—they result in a plausible probabilistic map for the location of each visual area (Bridge, 2011). On qualitative assessment, this localization of areas V1, V2, and V3 (ventral) also overlaps almost completely with probabilistic maps constructed using cytoarchitectonic, post-mortem definitions (Rottschy et al., 2007). Therefore we are confident that this mapping approach provides “ground truth” to the same extent as any currently available retinotopic mapping procedure in MRI.

### MEG-fMRI comparisons

#### Source localization accuracy

To evaluate MEG source localization accuracy relative to fMRI, we calculated the percentage of active vertices inside a particular visual cortical area that were localized to the retinotopically expected subregion of that area. The retinotopically expected subregion was defined in each individual, according to their fMRI-defined retinotopic map, and was evaluated for each stimulus. For example, to evaluate localization accuracy for an upper right (UR) quadrant in area V1, we calculated the percentage of active vertices within V1 that were localized into the left ventral subregion, which is the retinotopically expected location for that visual field stimulus. Localization accuracies for quadrant stimuli were averaged across stimuli and participants for each ROI. For rings, eccentricity band ROIs corresponding to stimulus eccentricities were defined across areas V1, V2, and V3 combined. Of the active vertices located across all the eccentricity bands, we calculated the percentage that localized into the retinotopically-expected band, separately for each cortical hemisphere, for each participant. The same procedure was used for quarter-rings, where retinotopic subregions were defined by both the angular visual field location and stimulus eccentricity.

As many of the resultant localization accuracy values were not normally distributed (MATLAB's Lillifors test, *p* < 0.05), non-parametric Wilcoxon signrank tests were used to calculate whether the sources were significantly localized into the retinotopically expected subregions (*p* < 0.05). For quadrants, chance level was 25% for visual areas V1, V2, and V3, for which we defined four angular subregions each (dorsal and ventral subregions in the left and right hemisphere), and 50% for V3A for which we defined two subregions only (left and right hemisphere). For rings, chance level was 33% as three eccentricity bands were defined. For quarter-rings, chance level for angular localization into the retinotopic quadrant was 25% and chance level for eccentricity localization into the eccentricity band within the quadrant was 33%.

Within each stimulus set, a Bonferroni multiple-comparison correction was applied to the statistical tests across visual areas and MEG reconstruction methods. A Kruskal-Wallis test was used to ascertain whether localization accuracy was different between source reconstruction methods.

If a source reconstruction approach resulted in no active vertices in the relevant early visual area for a particular stimulus and individual, this was excluded from the accuracy analyses. Aside from area V3A for quadrants (MNE: 16 rejections; beamformer: 12; MSP: 10; IID: 5; time-frequency beamformer: 14) and eccentricity reconstructions for lower-field quarter-rings (beamformer: 5 rejections), there were on average only 1 or 2 such failed localization per participant in each stimulus set for each source reconstruction method. We recalculated all results with the failed reconstructions included (*data not shown*); this slightly reduced overall localization accuracy rates, as expected, but did not affect the overall conclusions of the study.

#### Threshold for active vertices

The MEG source reconstruction methods output a statistical activity value, either F (MNE) or *t* (beamformer, MSP), for each point in the source space. This reflects that, in the context of source reconstruction algorithms, each potential source location has a probabilistic contribution to the MEG sensor signal because both noise in the data and the ill-posedness of the inverse problem preclude a unique, determined solution. A cut-off threshold must therefore be designated, which controls whether or not a particular cortical vertex is considered “active” in any given source reconstruction result. A non-systematic designation may affect the retinotopic MEG-fMRI comparisons in unexpected ways and thereby render unfair the comparison between reconstruction methods.

We defined the cut-off threshold in terms of the percentage of highest-responding vertices, across the cortex, that are designated “active”. For example, a threshold of 1% indicates that only vertices with activity values in the top 1% are designated “active”. We systematically calculated localization accuracy (as described above) as a function of cut-off threshold for each reconstruction method and visual stimulus, across visual areas V1, V2, and V3, individually for each participant. For a given threshold, if a stimulus resulted in zero “active” vertices in early visual areas for a particular participant, the accuracy result was set to zero. The optimal threshold was defined as the cut-off threshold which produced the most accurate source reconstructions for a given reconstruction approach. We then used this optimal threshold, set independently for each participant and for each reconstruction approach, in the localization accuracy evaluations and comparisons presented in the study. Optimal thresholds, converted to statistical values, for quadrant stimuli (averaged across participants) were *F* = 27.8 (MNE), *t* = 9.7 (beamformer for evoked response), *t* = 10.9 (beamformer for 7–9 Hz time-frequency window), *t* = 4.2 (MSP) and *t* = 2.1 (IID). For ring stimuli these were *F* = 17.5 (MNE), *t* = 10.8 (beamformer for evoked response) and *t* = 6.85 (MSP). For quarter-rings, average optimum thresholds across participants were *F* = 9.0 (MNE) and *t* = 8.3 (beamformer) for angular retinotopy, and *F* = 7.2 (MNE) and *t* = 7.7 (beamformer) for eccentricity mapping.

## Results

### Localization of visually evoked responses to angular retinotopic stimuli

**Figure 1:**
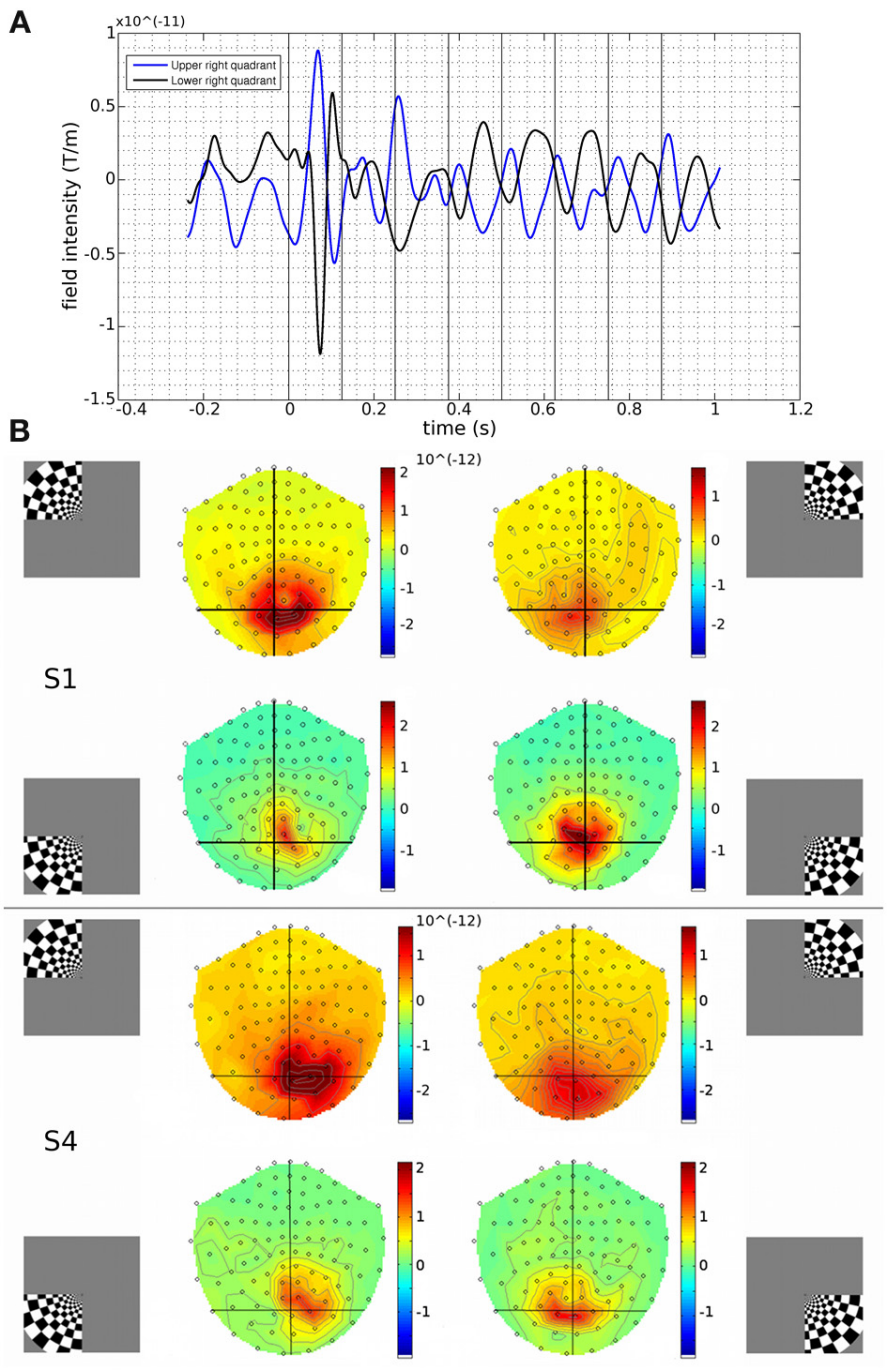
**(A)** Measured responses to upper and lower right quadrant stimuli, from a MEG channel located over the occipital cortex (gradiometer channel 1922), for Subject 1. Traces were time-locked to the onset of the visual stimulus (time = 0) and averaged. Changes to the stimulus contrast occurred every 125 ms following the onset of the visual stimulus (vertical black lines). Deflections of evoked responses to the upper and lower quadrants show opposite polarities, as might be expected from oppositely oriented current sources in the lower and upper calcarine banks respectively (Wandell et al., 2005). Source reconstructions were performed either on the visually evoked response (first response peak (FRP): centred at 83 ms) or upon the ongoing stimulus-induced oscillations at 8 Hz (200–1000 ms). **(B)** Gradiometer topographic maps (T/m) of averaged evoked responses at 83 ms post-stimulus for Subject 1 (S1) and Subject 4 (S4). Insets indicate stimulus locations. Black vertical and horizontal lines are presented to aid visualization. Peak responses tended to be over the hemisphere contralateral to the visual stimulus. Upper visual field stimuli evoked activation further back over the occipital pole than lower field stimuli.

Contrast-reversing checkerboard quadrant stimuli were presented to six human observers and the evoked brain responses were measured with MEG. Quadrants of visual stimulation were located in the upper left (UL), upper right (UR), lower right (LR) or lower left (LL) visual field. In response to stimulus onset, occipital and parietal MEG sensors showed large deflections at 60–100 ms. Subsequent responses to the contrast reversals of the stimulus are seen throughout the stimulus duration (Figure 1A). Scalp topography of MEG gradiometer sensor activity shows how responses vary by stimulus location, roughly according to the expected retinotopic pattern (Figure 1B).

**Figure 2:**
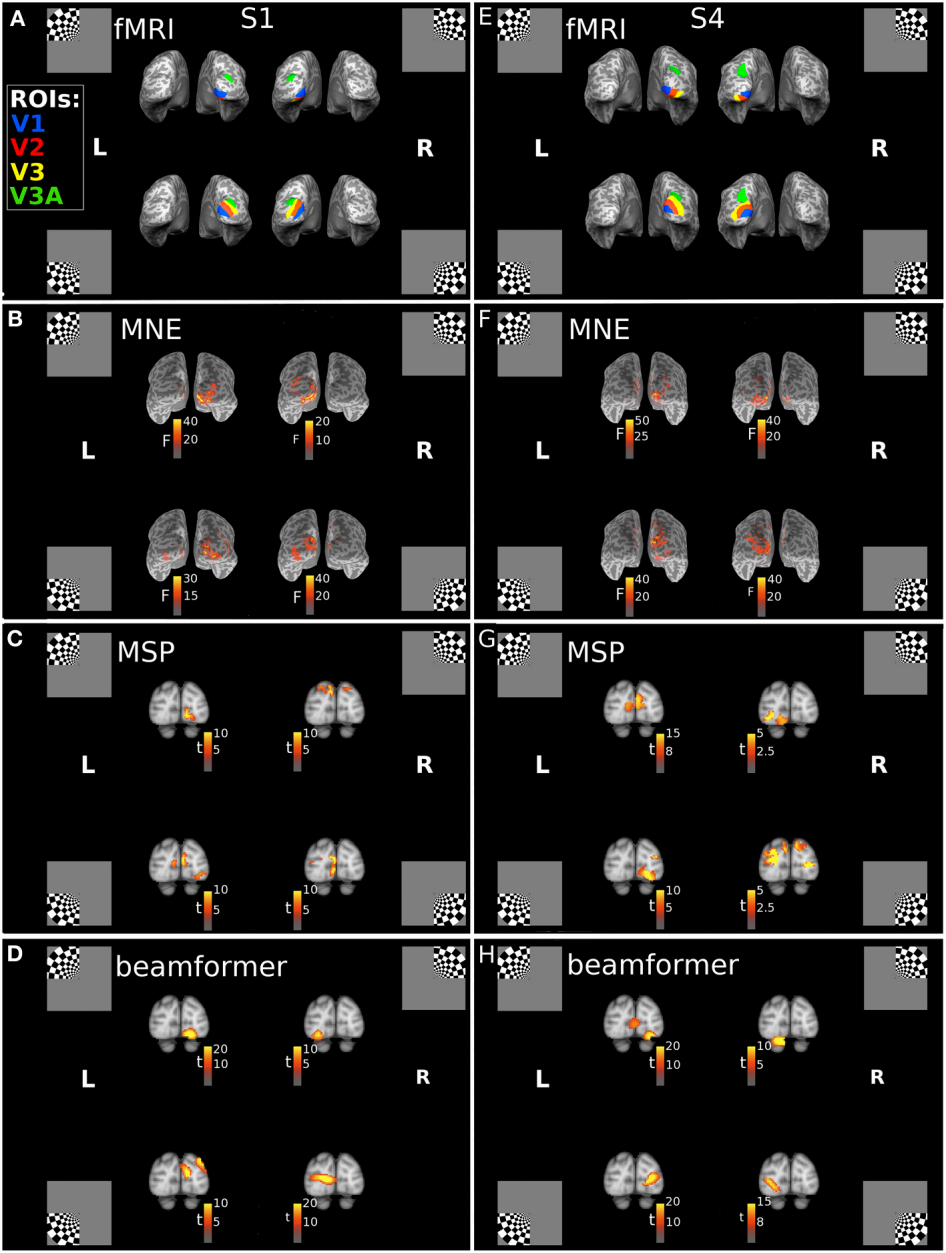
**Locations of the fMRI- and MEG-measured brain responses to quadrant stimuli. (A,E)** Individual fMRI-defined ventral and dorsal subregions of early visual areas, which respectively represent upper field and lower field quadrant stimuli, for Subject 1 (S1) and Subject 4 (S4). Blue = area V1; Red = area V2; Yellow = area V3; Green = area V3A. Insets indicate stimulus locations. **(B–D)** Retinotopic source reconstructions of MEG responses to quadrant stimuli around the first response peak (FRP) for Subject 1. **(F–H)** Retinotopic Source reconstructions of MEG responses to quadrant stimuli around the first response peak (FRP) for Subject 4. F-statistic results for the minimum norm estimate approach (MNE), calculated using pre-stimulus variance estimates, are plotted on the individuals' inflated cortical surface **(B,F)**. The t-statistic results for the multiple sparse priors (MSP) approach **(C,G)** and beamformer **(D,H)**, calculated with a contrast of responses across stimuli, are displayed volumetrically on the MNI152 template brain.

Cortical sources of the first response peak (FRP) of the visually evoked response were localized with three reconstruction approaches: minimum norm estimate (MNE), beamformer (BF), and multiple sparse priors (MSP). Each of these approaches incorporates a different set of prior assumptions to solve the inverse problem of source reconstruction. We found that the MNE approach consistently localized sources of the MEG signals to the hemisphere contralateral to the quadrant location. A consistent dorsal-ventral distinction for lower and upper field stimuli was also present, in line with the pattern expected from the individuals' fMRI-defined retinotopic maps (Figures 2A,B,E,F). The multiple sparse priors (MSP) and beamformer approaches both resulted in localizations that generally followed this retinotopic pattern with some deviations (Figures 2C,D,G,H). For example, the MSP reconstruction of responses to the UR quadrant of Subject 1 (Figure 2C) localized sources to the dorsal occipital lobes, instead of the ventral left lobe.

Localization accuracy of each reconstruction approach was evaluated by assuming that the fMRI retinotopic map is a gold-standard, and by calculating the percentage of active cortical vertices localized into the retinotopically-defined subregion of each early visual area (V1, V2, V3, V3A) for each participant. These percentages were calculated for each participant based on their own fMRI-defined map and were then averaged over stimuli and participants. We systematically calculated the localization accuracy as a function of the cut-off threshold for including active vertices into the analysis for each reconstruction method and visual stimulus, across visual areas V1, V2, and V3, individually for each participant (see Methods, Threshold for active vertices). The optimal threshold that produced the most accurate source reconstructions for a given participants and method was used.

**Figure 3:**
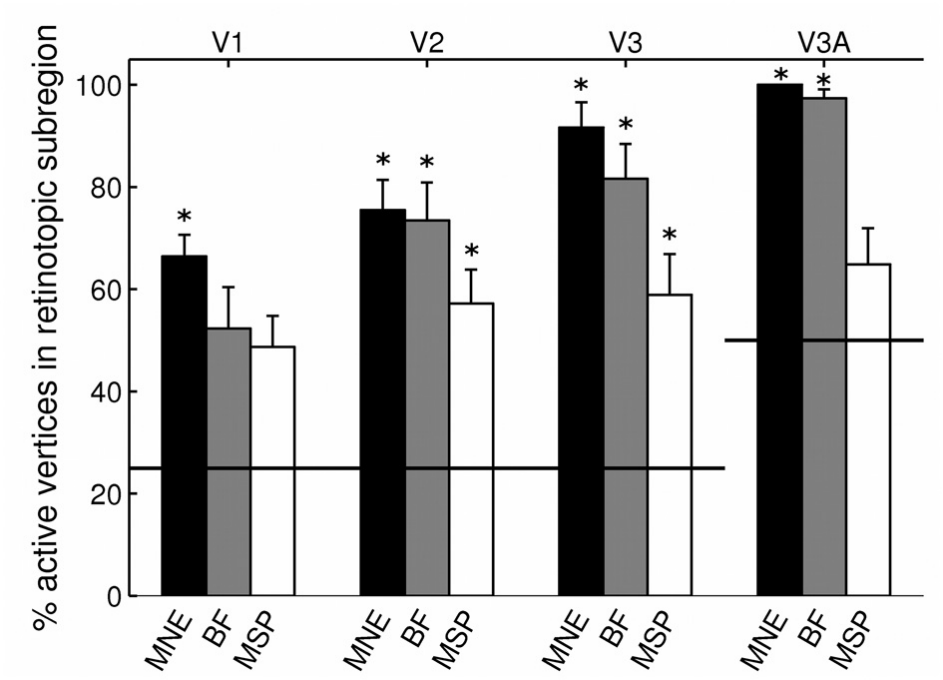
**Source localization accuracy of evoked responses to quadrant stimuli, reconstructed with minimum norm estimate (MNE, black), beamformer (BF, gray), and multiple sparse priors (MSP, white)**. Bars show percentage of active vertices localized to the fMRI-defined subregion of each of four early visual areas (V1, V2, V3, and V3A). Error bars show s.e.m. Black lines indicate chance accuracy level for each early visual area ROI. ^*^indicates *p*_*m*_ < 0.0031 for Wilcoxon signrank test of localization accuracy compared to chance (*p*_*i*_ < 0.05 with Bonferroni correction for 16 multiple comparisons).

Angular retinotopic localization accuracy measured in this way was significant for all four early visual areas for MNE, for 3 of 4 visual areas with beamforming, and for 2 of 4 visual areas with MSP (Wilcoxon signrank tests: *p* < 0.05 Bonferroni corrected for multiple comparisons; Figure 3, Table 2). On average, MNE was most successful in localizing the highest percentage of active vertices to the expected retinotopic subregions (mean areas V1–V3 combined: 77.9%; V3A: 100%), followed by the beamformer (mean areas V1–V3 combined: 69.1%; V3A: 97.4%), followed by MSP (mean V1–V3: 54.9%; V3A: 64.9%). Localization accuracies of the three reconstruction methods were significantly different (Kruskal-Wallis tests: areas V1–V3 combined: chi^2^ = 18.4, *p* < 0.001; V3A, chi^2^ = 14.9, *p* < 0.001).

**Table 2:** **Localization accuracy for angular mapping**.

**ROI (chance (level)**	**MNE**	**Beamformer (evoked response)**	**Beamformer (time-frequency)**	**MSP**
V1 (25%)	66.4 (±20.8)	52.3 (±39.6)	68.6 (±33.6)	48.7 (±29.9)
V2 (25%)	75.5 (±29.1)	73.5 (±36.3)	68.2 (±39.9)	57.2 (±32.5)
V3 (25%)	91.7 (±24.2)	81.6 (±33.5)	61.3 (±44.3)	58.9 (±39.1)
mean (V1, V2, V3)	77.9	69.1	66.0	54.9
V3A (50%)	100.0 (±0)	97.4 (±8.6)	83.2 (±36.2)	64.9 (±34.8)

*Percentage of active vertices that localized to the fMRI-defined subregion of individuals' retinotopic maps for each early visual area, for quadrant stimuli. Results, averaged across stimuli and participants (±SD), are shown for MNE, beamformer (evoked and time-frequency approaches) and MSP reconstruction methods*.

To further investigate the factors contributing to the different localization accuracy values for minimum norm vs. multiple sparse priors, source reconstruction was carried out in the SPM8 software using the IID (independently and identically distributed priors) source reconstruction. The IID algorithm corresponds to a minimum norm assumption, but does not incorporate the same depth weighting and anatomical constraints as the MNE software. IID localization accuracy was better than chance for all 4 visual areas tested and the mean accuracy values were slightly higher for IID than MSP (IID: mean areas V1–V3 combined: 57.3%; V3A: 84.8%). However, this difference was not significant (Kruskall-Wallis: areas V1–V3: chi^2^ = 0.1, *p* = 0.75, area V3A: chi^2^ = 3.58; *p* = 0.058). This suggests that the depth weighting and anatomical constraints of the MNE implementation convey some advantage for retinotopic mapping. Increasing the time window to capture a wider section of the visually evoked response did not improve the MSP localization accuracy for angular retinotopy (see Table 1).

### Beamforming source reconstruction of stimulus-induced oscillations

Visual stimuli underwent contrast reversal at a rate of 4 Hz, evoking ongoing oscillations in brain responses at a rate of 8 Hz (Figure 1A). A time-frequency (TF) beamformer was focused on the 7–9 Hz frequency band of measured brain responses, 200–1000 ms post-stimulus onset. This excluded the first response peak (FRP) of the MEG response. Beamformer localization accuracy was similar regardless of whether the FRP (described in Localization of visually evoked responses to angular retinotopic stimuli) or the 7–9 Hz frequency band signals were used (time-frequency beamformer: mean areas V1–V3 combined: 66.0%; V3A: 83.2%; Kruskall-Wallis: areas V1–V3: chi^2^ = 0.18, *p* = 0.67, area V3A: chi^2^ = 0.15; *p* = 0.70; Table 2). Both approaches resulted in localization at a level significantly better than chance for 3 of the 4 early visual areas tested, although the regions that failed to reach significance were different for the two approaches. The use of the stimulus frequency tag therefore resulted in source localizations that were as good as, but not significantly better than, the application of the beamformer to the FRP.

### Localization of visually evoked responses to eccentricity-varying stimuli

To investigate whether retinotopic localizations could be obtained for eccentricity-varying stimuli as well as for angular stimuli, brain responses to contrast-reversing concentric rings were measured with MEG in seven participants. Three ring eccentricities were used: ECC 1 (0–0.75°), ECC 2 (1.0–2.0°), and ECC 3 (3.0–5.4°).

**Figure 4:**
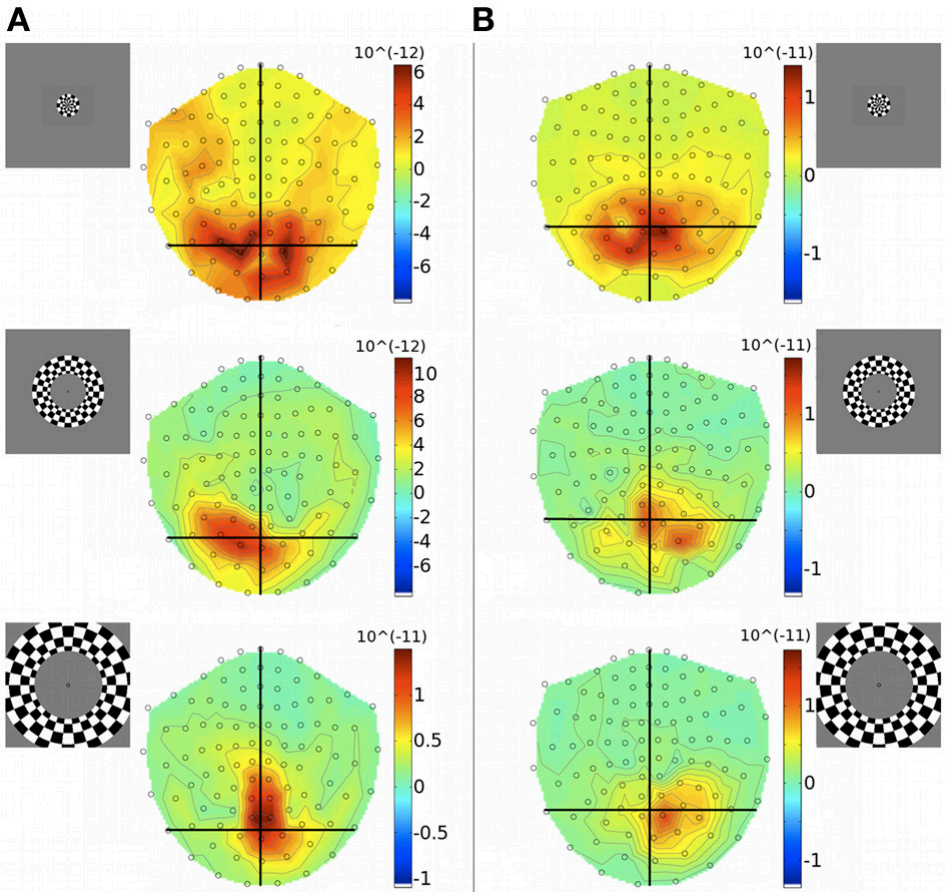
**Gradiometer topography maps (T/m) of the averaged evoked responses to concentric ring stimuli at 83 ms post-stimulus, for Subjects 1 (A) and 4 (B)**. Eccentricities ECC 1, ECC 2, and ECC 3 are presented at the top, middle, and bottom, respectively. Insets indicate stimulus locations.

Unlike quadrants, rings were bilateral stimuli, extending over both halves of the visual field and were expected to activate both cortical hemispheres simultaneously. At the level of MEG sensor topography, evoked responses to rings did not show a clear spatial pattern according to stimulus eccentricity and some responses appeared unilaterally biased (Figure 4).

**Figure 5:**
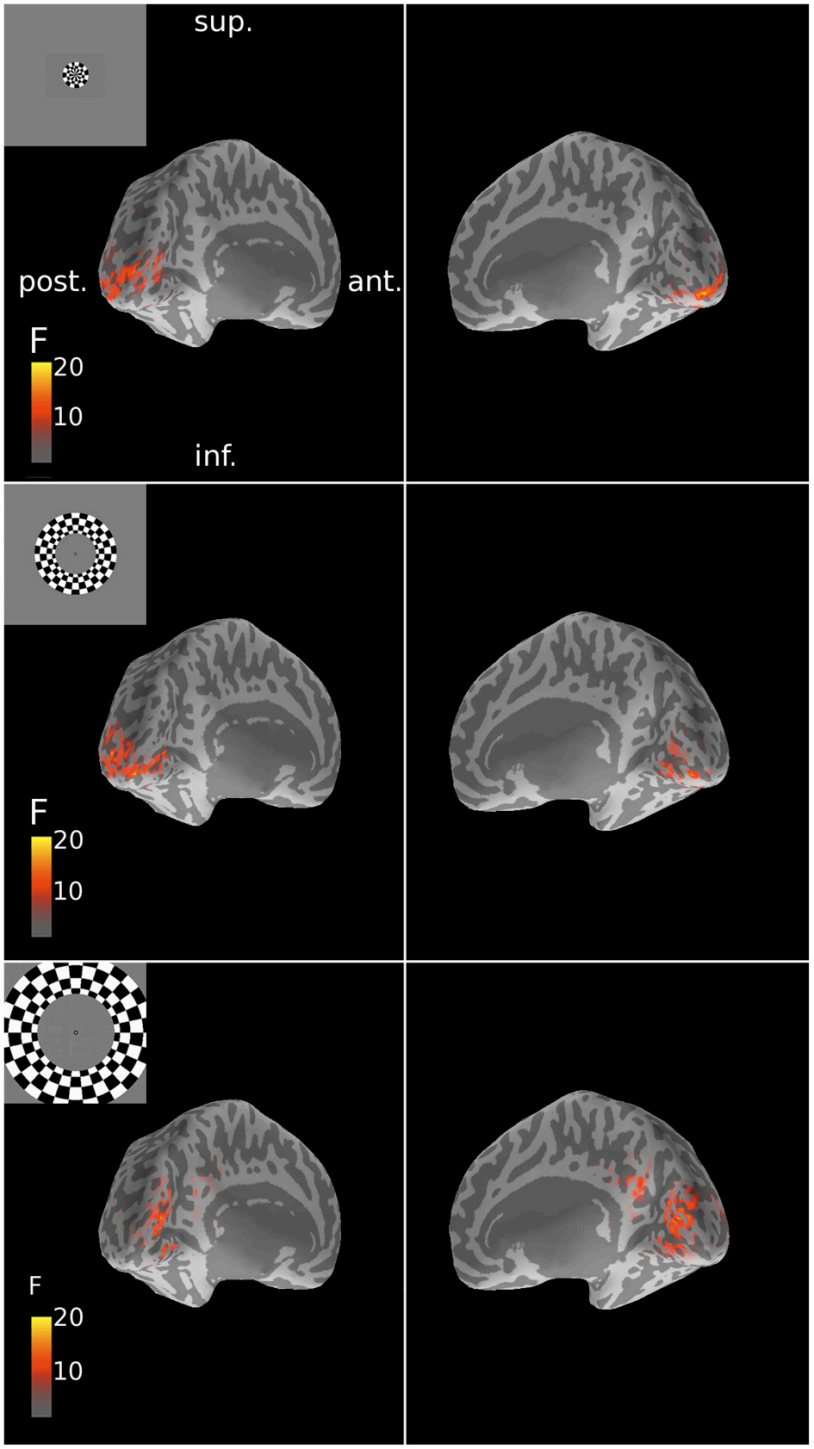
**Spatial patterns of MNE source reconstructions of responses to ring stimuli for Subject 6**. These reconstructions followed the expected retinotopic posterior-anterior progression with increasing stimulus eccentricity. F-statistic results are presented for left and right inflated cortical surfaces (see Figure 1). Insets show the corresponding stimulus locations. Sup., superior; post., posterior; ant., anterior; inf., inferior.

**Figure 6:**
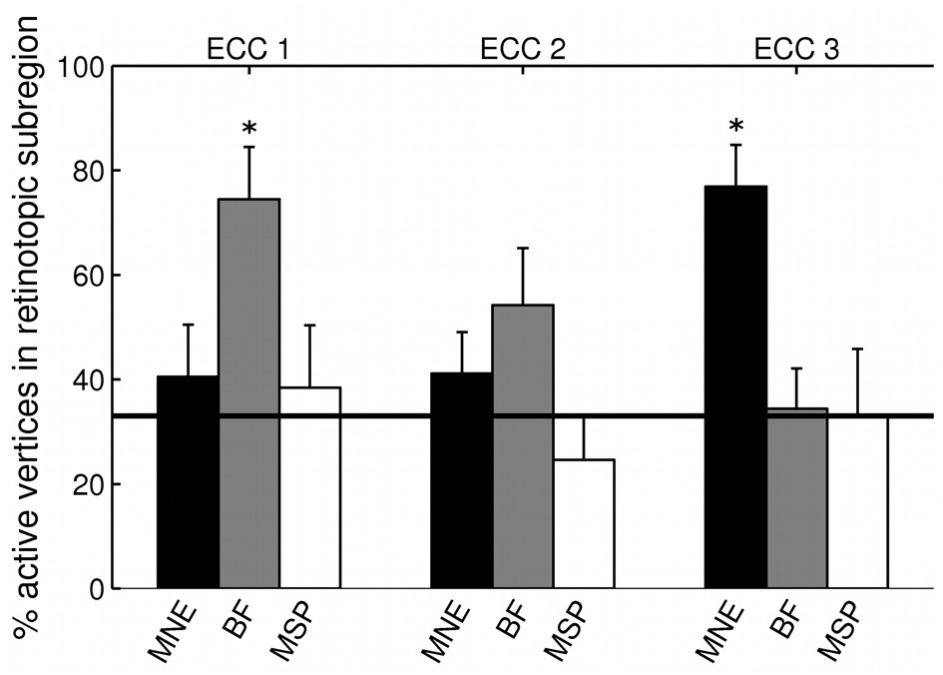
**Source localization accuracy of evoked responses to ring stimuli, reconstructed with MNE (black), beamformer (BF, gray), and MSP (white)**. Bars show percentage of active vertices localized to the fMRI-defined eccentricity band across visual areas V1, V2, and V3 for each stimulus eccentricity (ECC 1, ECC 2, ECC 3). Error bars show s.e.m. Black line indicates chance accuracy level (33%). ^*^indicates *p*_*m*_ < 0.0056 for Wilcoxon signrank test of localization accuracy compared to chance (*p*_*i*_ < 0.05 with Bonferroni correction for 9 multiple comparisons).

The three MEG source reconstruction approaches were used to reconstruct sources of the visually evoked responses to ring stimuli. Localization accuracy of responses to each type of ring stimulus into the individual participant's fMRI-defined eccentricity band was evaluated across visual areas V1, V2, and V3 combined, then averaged across participants. For Subject 6, the minimum norm estimate (MNE) reconstruction of sources to concentric rings followed the expected posterior-anterior progression in the early visual areas of the calcarine region as stimulus eccentricity increased (Figure 5). However, this result was the exception; retinotopic sources for responses to rings were not consistently obtained across the other six participants with any reconstruction approach. Localization accuracy was not significantly better than chance for ECC 2 stimulus responses for any source reconstruction method (Figure 6, Table 3). The minimum norm approach (MNE) localized sources accurately to the expected eccentricity band for ECC 3 stimuli and the beamformer for ECC 1 stimuli (Figure 6). The accuracies of the three reconstruction methods were significantly different from each other (Kruskal-Wallis: chi^2^ = 10.7, *p* < 0.01).

**Table 3:** **Localization accuracy for eccentricity mapping**.

**Ring eccentricity (chance level)**	**MNE**	**Beamformer (evoked response)**	**MSP**
ECC 1 (33%)	40.5 (±31.6)	74.5 (±31.7)	38.4 (±37.7)
ECC 2 (33%)	40.1 (±41.1)	54.2 (±34.6)	24.6 (±26.5)
ECC 3 (33%)	76.9 (±25.2)	34.3 (±24.4)	33.0 (±24.4)
Mean	52.5	54.3	32.0

*Percentage of active vertices that localized into the fMRI-defined subregion of individuals' retinotopic maps across early visual areas V1, V2, and V3, for ring stimuli. Results, averaged across stimuli and participants (±SD), are shown for MNE, beamformer, and MSP reconstruction methods*.

### Effect of confining eccentricity-varying stimuli to a visual field quadrant

**Figure 7:**
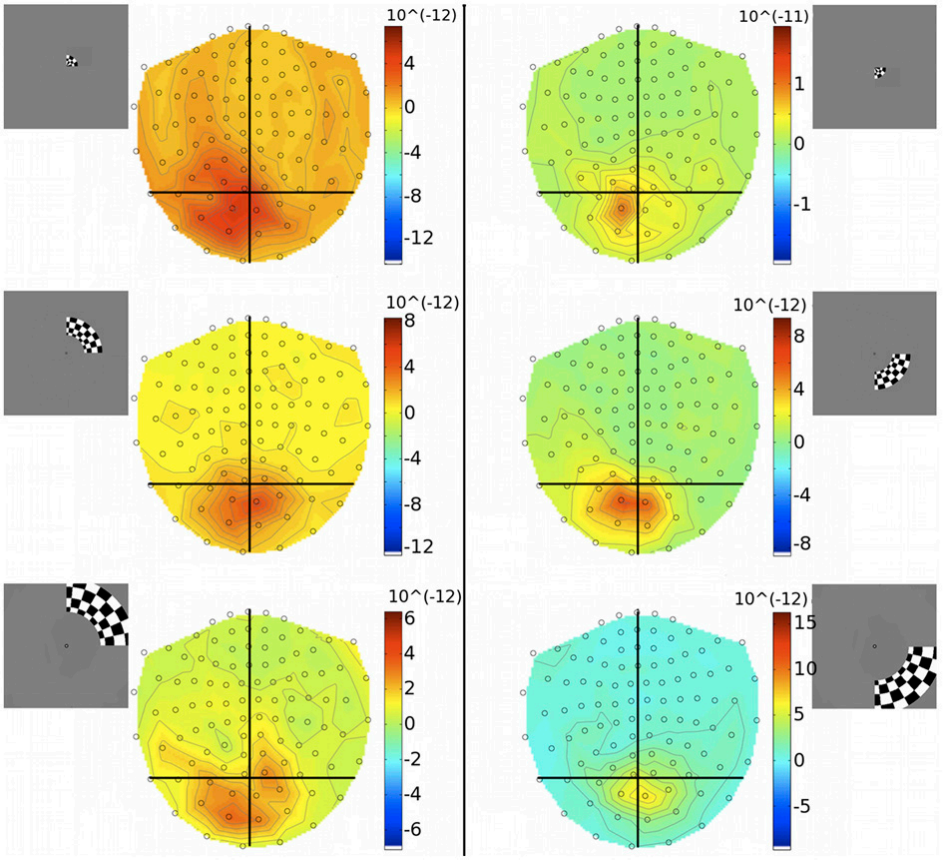
**Gradiometer topography maps (T/m) of the averaged evoked response to quarter-ring stimuli at 83 ms post-stimulus (Subject 2)**. **Left panels:** show the responses to upper field quarter-ring stimuli. Quarter-ring eccentricities U-ECC 1, U-ECC 2, and U-ECC 3 are presented at the top, middle, and bottom, respectively. **Right panels:** show the responses to lower field quarter-ring stimuli. Quarter-ring eccentricities L-ECC1, L-ECC2, and L-ECC3 are presented at the top, middle, and bottom, respectively. Insets indicate stimulus locations.

To investigate the discrepancy in the success of retinotopic localization of visual responses to quadrants vs. concentric rings, five of the seven participants who were scanned with eccentricity-varying stimuli were re-scanned with an amended stimulus set (quarter-rings), which consisted of the checkerboard ring stimuli confined to either the upper or lower quadrant of the right visual hemifield. Quarter-rings were located within the same eccentricity bands (and hence retinotopic brain representations) as the corresponding ring stimuli. Therefore, there were 6 quarter-ring stimuli: three presented in the upper right visual field quadrant (U-ECC 1, U-ECC 2, U-ECC 3) and three presented in the lower right visual field quadrant (L-ECC 1, L-ECC 2, L-ECC 3). MEG sensor topographies show that activations tend to lie over the left cortical hemisphere, as expected from angular retinotopy, but again no clear topography by eccentricity is discernible (Figure 7). We evaluated localization accuracy of sources of brain responses to quarter-rings reconstructed by minimum norm estimates and the beamformer for evoked responses. MSP reconstructions did not consistently localize activity into the early visual areas for any participant (*data not shown*), so we focus on MNE and the beamformer in the present analysis.

#### Angular retinotopy with quarter-rings

**Figure 8:**
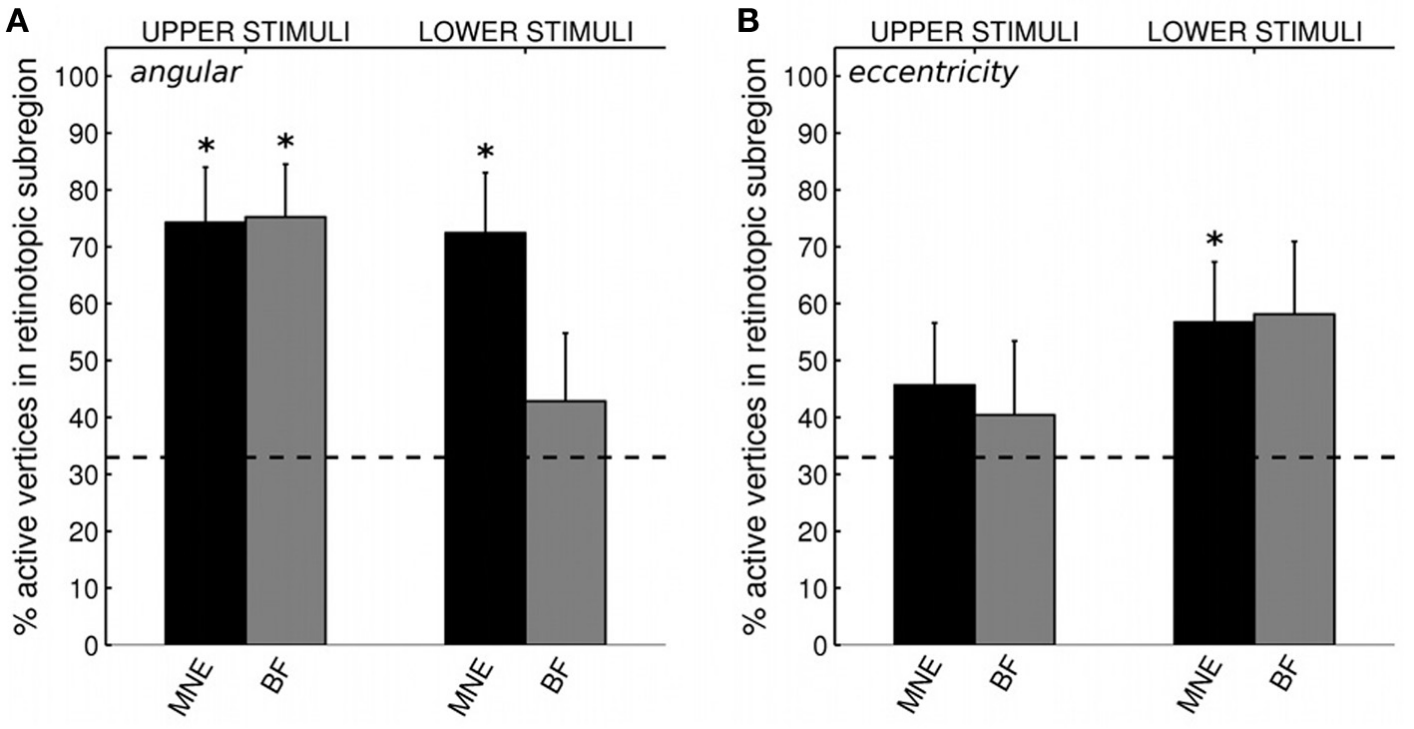
**(A)** Source localization accuracy, according to angular retinotopy, of evoked responses to quarter-ring stimuli with MNE (black) and beamformer (BF, gray) methods. Bars show percentage of active vertices localized to the fMRI-defined retinotopic subregion across early visual areas V1, V2, and V3. Error bars show s.e.m. Black dashed line indicates chance accuracy level for each early visual area ROI. ^*^indicates *p*_*m*_ < 0.0125 for Wilcoxon signrank test of localization accuracy compared to chance (*p*_*i*_ < 0.05 with Bonferroni correction for 4 multiple comparisons). **(B)** Source localization accuracy, according to eccentricity, of evoked responses to quarter-ring stimuli, with MNE (black) and beamformer (BF, gray). Bars show the percentage of active vertices localized to the corresponding fMRI-defined eccentricity band, considered within the corresponding angular subregion of early visual areas V1, V2, and V3. Error bars and statistical comparisons as for **(A)**.

We first confirmed that brain responses to quarter-rings were adequately mapped according to angular retinotopy, by calculating the percentage of active vertices localized into the expected subregion of early visual areas V1, V2, and V3, combined together. For example, brain sources of responses to upper field quarter-ring stimuli are expected to localize to the left ventral subregion, whilst lower field quarter-ring stimuli to the left dorsal subregion. Results are presented separately for upper or lower visual field locations, averaged over stimuli and participants (Figure 8A). Reconstructing sources with the minimum norm approach (MNE) resulted in both upper and lower field stimuli sources localized into the fMRI-defined quadrant subregion at levels better than chance (Wilcoxon signrank: *p* < 0.001). Localization accuracy was comparable to that of quadrant stimuli reported with the MNE method above (mean over all stimuli: 73.3%, Table 4). For the beamformer, responses to upper field stimuli were well localized according to angular retinotopy (*p* < 0.001; mean: 75.4%) whilst responses to lower field stimuli were not localized better than chance level to the expected dorsal subregions (mean: 42.9%, *p* = 0.090, *n* = 15; Table 4). Beamformer reconstructions were however mapped according to angular retinotopy at a level better than chance when considered over all stimuli (mean over all stimuli: 59.0%, *p* < 0.001, *n* = 30).

**Table 4:** **Localization accuracy for quarter-ring stimuli**.

	**MNE**	**Beamformer**
**ANGULAR LOCALIZATION (CHANCE LEVEL 25%)**		
Upper field stimuli	74.3 (±30.1)	75.4(±29.3)
Lower field stimuli	72.4 (±33.4)	42.9 (±37.9)
Combined mean	73.3	59.0
**ECCENTRICITY LOCALIZATION (CHANCE LEVEL 33%)**		
Upper field stimuli	45.7 (±34.4)	40.4 (±41.0)
Lower field stimuli	56.8 (±33.5)	58.2 (±40.5)
Combined mean	51.4	49.0

*Percentage of active vertices in early visual cortex that localized into the fMRI-defined subregion of individuals' retinotopic maps, for quarter-ring stimuli. Results are reported for upper or lower visual field and averaged across stimuli and participants (±SD) according to either angular (expected quadrant) or eccentricity retinotopy (expected eccentricity band)*.

#### Eccentricity localization with quarter-rings

We then evaluated the localization accuracy of responses to quarter-ring stimuli into the expected eccentricity band (within the expected angular retinotopic cortical subregion). Although mean localization accuracy values were numerically similar for both reconstruction methods, accuracy was significantly better than chance for the MNE method (mean across all stimuli: 51.4%; Wilcoxon signrank test: *p* < 0.01, *n* = 14) but did not reach significance for the beamformer (mean across all stimuli: 49.0%; Wilcoxon signrank test: *p* = 0.065, *n* = 10). On average, localization accuracy values were higher for lower visual field stimuli (mean across methods: 57.5%) than for upper visual field stimuli (mean across methods: 43.1%), but this difference did not reach significance (Kruskal-Wallis: chi^2^ = 3.42, *p* = 0.064; Table 4). Only MNE reconstructions of brain responses to lower field stimuli were significantly better than chance when considered on their own (Figure 8B). Average accuracy values were close to those obtained for ring stimuli but not as high as those for angular retinotopy (quadrant stimuli) for the same reconstruction approaches (Table 4).

## Discussion

### Source localization accuracy of visual responses to stimuli varying by angular location

Minimum norm estimate (MNE), beamformer, and multiple sparse priors (MSP) source reconstruction methods were used to reconstruct sources of visual brain responses to angular retinotopic stimuli (quadrants). Source localization accuracy was defined by how accurately the different MEG reconstruction methods could match fMRI retinotopic maps for each individual. On average, localization accuracy was higher for MNE source reconstructions than for the beamformer, which in turn was higher than MSP. The MNE approach assumes that source amplitudes are minimal whilst brain sources are many and independently distributed (Dale et al., 2000; Gramfort et al., 2014). Our results show that this approach produces—in conjunction with specific depth-weighting and anatomical constraints—reliable source reconstructions of retinotopic activity in early visual cortex.

The beamformer, on the other hand, uses a spatial filtering algorithm to estimate the time course of activity at each brain source independently (van Veen et al., 1997; Hillebrand and Barnes, 2011). The difference in localization accuracy between MNE and beamformer may be due to the different reconstruction algorithm. However, the MNE and beamformer methods implemented in the analysis packages used here additionally differ in their utilization of anatomical information; MNE uses the individual's cortical surface as the source space for reconstruction and hence imposes an additional constraint on the solution of the inverse problem, whilst the beamformer evaluates signals independently throughout the cranial volume (see Table 1). Lack of a cortical anatomical prior may have contributed to the lower spatial resolution of the beamformer compared to MNE.

The multiple sparse priors (MSP) approach showed a trend of localizing sources to the expected angular subregions of early visual areas, but this reached significance only in areas V2 and V3. On average, localization accuracy was lower for MSP than for MNE and the beamformer. This was surprising, as a previous study had shown adequate source localizations of responses to visual face stimuli with the MSP assumptions, with results superior to those of SPM8's minimum norm implementation (IID) when goodness of reconstruction was evaluated by Bayesian model evidence (Henson et al., 2009). The MSP assumptions may have worked well when applied to brain responses to faces because the expected responding regions (fusiform face areas) are large, bilateral clusters, matching the MSP prior assumptions of sparseness and bilateral components, based upon functional anatomy, which are selected by the algorithm according to the correlations present in the data (Friston et al., 2008). However, this pattern may not adequately reconstruct brain activity patterns for the angular retinotopy stimuli used in this study, which are biased unilaterally and spread irregularly over occipital areas in different individuals. Our finding is in line with a recent result of Cottereau et al. (2012) in their evaluation of the use of fMRI maps as spatial *priors* for source reconstruction of simulated MEG data arising from early visual sources. Cottereau et al. evaluated source reconstructions by calculating both the focalization error (the ratio between the estimated and theoretical energies of the current at the simulated sources) and the relative energy (the ratio between the normalized energies contained in the estimation of the active sources and the global distribution). They report that although the MSP approach had slightly better relative energy estimates, it also had higher focalization errors when compared to the minimum norm (MNE equivalent).

The source space used for MSP reconstruction in the SPM8 software was the inverse-normalized template cortical mesh (Mattout et al., 2007; Henson et al., 2009; Litvak et al., 2011), rather than the individual's cortical template, which was used for MNE (see Table 1). A key advantage of this approach is it that it facilitates group level analysis and also facilitates the inclusion of fMRI priors for MEG analysis, which are typically defined in the template space. Previous studies have demonstrated that source reconstruction of evoked responses is not impaired by the use of the inverse-normalized template rather than the individual's cortical mesh (Mattout et al., 2007; Henson et al., 2009). This suggests that it is the assumptions of multiple sparse priors that underlie the difference in source localization accuracy between the MSP and the MNE methods. An alternative approach within the SPM8 software is IID (independent and identically distributed sources), which corresponds to minimum norm assumptions. Implementation of IID on the same MEG data in SPM8 software gives localization accuracy better than chance for all four early visual areas, compared to just two early visual areas for MSP.

However, mean localization accuracy values were not significantly different between IID and MSP and were generally lower for IID than for the MNE approach. This may be due to further differences between IID and MNE implementations, such as the use of depth-weighting in MNE to counteract the superficial bias of minimum norm assumptions (Lin et al., 2006). It could also be due to the differences in use of anatomical data for source space specification. Variability in individuals' cortical surface geometry around the tightly folded early visual areas may mean that the use of the individual's mesh rather than the inverse-normalized cortical template mesh makes a significant contribution to accurate localization of responses in experiments investigating the visual system. This could also apply to other tightly folded brain regions. Future updates to the IID and MSP reconstruction algorithms could include the option to utilize the individual's cortical surface, rather than the inverse-normalized template, for source space modeling.

Cottereau et al. (2011) reconstructed retinotopic sources accurately into early visual areas V1, V2, and V3, by using a stimulus contrast reversal frequency tag. We tested whether use of an ongoing frequency tag may be an improvement on using the first response peak (FRP). With the beamformer, we found similar retinotopic localization accuracy when analyzing source power at the second harmonic of the stimulus contrast reversal frequency as compared to the reconstruction of FRP. In both cases, the accuracy localizations were significantly better than chance for 3 of the 4 early visual areas. Cottereau et al. (2011) used a faster stimulus contrast-reversal rate (7.5 Hz; second harmonic: 15 Hz) and a wider time window for source reconstruction (5600 ms), such that they focused on localizing a “steady state” visual response. In the current study, stimulus contrast-reversal rate was 4 Hz (second harmonic: 8 Hz) and the time window was 800 ms long, perhaps resulting in a noisier power estimate that might have limited the localization accuracy. Therefore, a steady-state response longer than the one utilized in the present study may be necessary for the frequency-tag information to improve source reconstruction.

### Source localization accuracy of visual responses to stimuli varying by eccentricity

Concentric rings are commonly used to map eccentricity in early visual areas with fMRI (DeYoe et al., 1994; Engel et al., 1994; Wandell, 1999). None of the reconstruction methods consistently localized responses to the appropriate eccentricity bands in early visual areas at a level better than chance. This was unexpected, especially for MNE and beamformer approaches, which had reliably localized visual responses to angular retinotopy. Bilateral, eccentricity-varying visual stimuli may present a unique set of challenges to MEG source reconstruction. Concentric rings are full-field visual stimuli and so are expected to synchronously activate both upper and lower calcarine banks in both the left and the right cortical hemispheres, which may result in some interference or cancelation of equal and opposite magnetic fields arising from opposing cortical surfaces. Moreover, spatially extended and correlated source activity cannot be spatially filtered by the beamformer as easily (Hansen et al., 2010). Assumptions of multiple sparse priors (MSP) might have been expected to be more appropriate for localizing ring stimuli as they incorporate priors of bilaterality; however, this was not found to be the case.

To evaluate whether the bilateral extent of the ring stimuli limited the retinotopic localization by eccentricity, MEG signals were recorded with “quarter-rings” stimuli, which were confined to either the upper or lower quadrant of the right visual field. With MNE and beamformer approaches, the corresponding brain sources were generally well localized according to angular retinotopy. But with regard to localization to the expected eccentricity band, average accuracy values remained low, close to those obtained for whole rings. The MNE reconstruction method localized sources at a level better than chance when considered overall quarter-ring stimuli and for lower field stimuli alone.

There are a number of reasons that may explain the limitations of MEG source localizations to eccentricity stimuli of varying sizes. The representation of eccentricity in early visual areas varies along the lateral-medial and posterior-anterior axes of the brain, such that foveal stimuli are represented at the occipital pole and the representation of more peripheral locations progresses medially and anteriorly along the banks of the calcarine sulcus (Wandell et al., 2005). As a result, the greatest changes in MEG signal amplitude by stimulus eccentricity may occur in the same sensors due to nearer vs. deeper sources. By contrast, quadrant stimuli are represented in different hemispheres and may be expected to activate quite different sets of sensors. Indeed, when inspecting sensor topography, no consistent pattern could be seen by stimulus eccentricity, although this could be seen for angular stimuli. In MEG source reconstruction, there is inherent ambiguity in discerning low-amplitude superficial activity from higher-amplitude deep activity, which might explain the poor eccentricity results found here.

Sharon et al. (2007) used the MNE reconstruction approach to localize MEG responses to visual stimuli according to both angular and eccentric retinotopic position in occipital cortex. Their visual stimuli were small Gabor patches constructed from Gaussians of 1.2 or 1.7° full-width at half-maximum, thereby similar in extent to our quarter-ring stimuli U-ECC 1/L-ECC 1 (radius 0.75°) and U-ECC 2/L-ECC 2 (radius 1.0°). Sharon et al. defined localization error as the mean distance in the 3D volume between the centers-of-mass of the MEG and fMRI activity clusters. For the reconstruction of MEG signals alone, the localization error over six participants was found to be approximately 10 mm. While in their Figures 2, 3, the localization of MEG responses alone are mostly associated with the correct bank of the calcarine, the example MEG sources in Figure 2 do not unambiguously show the expected progression anteriorly or medially according to eccentricity. Only analysis of the centers of gravity of the source localizations show a slight trend to vary with stimulus eccentricity in the expected retinotopic pattern. As the radii of our quarter-ring stimuli were of similar magnitude to those of Sharon et al. (2007), it seems unlikely that size alone can account for any discrepancy in localization between the two studies. On the other hand, Sharon et al. (2007) presented each stimulus to viewers in a total of 500 trials, rather than the 95–125 trials in the present study. It may be therefore be that a much greater signal to noise ratio obtained by averaging over a much larger number of trials is necessary to successfully localize MEG signals by eccentricity, compared with angular retinotopy.

### Limitations

Localization accuracy of the different MEG analysis methods was evaluated by calculating what percentage of the active vertices in early visual areas V1, V2, V3, and V3A were located in the expected subregion according to fMRI retinotopy in the same individuals (see also Cottereau et al., 2011; Supplementary Data). We ignored the incidence of active vertices in areas such as LO, V4, V3B, hMT+, which were outside the areas studied here. An alternative way to test localization accuracy would be to calculate the percentage of cortical vertices, in a retinotopically expected subregion, that are “active” in response to the corresponding stimulus, relative to the total number of vertices in that subregion. However, this value would be difficult to interpret even with perfect MEG source reconstruction, because MEG sensors are blind to sources located at certain parts of the cortex, such as the crests of gyri, due to the geometry of magnetic fields of the brain relative to the orientation of the sensor array (Hansen et al., 2010). Nevertheless, future attempts at an anatomically corrected analysis of this type would be interesting. Alternatively, it would be possible to compare MEG and fMRI source localizations in the 3D volume, for example by computing the distance between the center of mass of the fMRI and the active vertices in the MEG source result (e.g., Sharon et al., 2007). We decided against this method because, for the large visual stimuli used here, this approach would not utilize all of the information available from the fMRI maps and a few peak responding vertices would not be indicative of the entire reconstruction result. Additionally, this measure of localization can be misleading for anything other than point stimuli.

An important assumption of this study was that fMRI retinotopy correctly localizes the true sources of brain responses in individual participants. Although there is a wealth of histological and lesion evidence to suggest that retinotopic mapping measured by fMRI corresponds to the true patterns (Holmes, 1918, 1945; Horton and Hoyt, 1991; Bridge et al., 2005; Bridge and Clare, 2006), there may be unknown differences between the exact locations of the sources of brain activity measured by MEG and fMRI. These methods detect different underlying processes (electrophysiological vs. metabolic) and the time-scale on which these processes change is different (milliseconds vs. seconds). MEG signals most likely arise from synchronous synaptic current in cortical pyramidal cells (Hämäläinen et al., 1993). There is ongoing research into the electrophysiological correlates of the fMRI BOLD signal but it seems to be linked to the local field potential (LFP), which is also a measure of total of synaptic activity in cortical cells (Logothetis et al., 2001). However, early visual cortex, especially striate cortex, is well perfused and blood vessels are likely to be spatially close to their neuronal sources (Engel et al., 1997). In all, these arguments indicate that an fMRI-MEG comparison is appropriate for evaluating MEG localization accuracy.

Some localization error must necessarily arise from inaccuracies in the source space specification from the anatomical MRI and its co-registration to the MEG coordinate space (Hillebrand and Barnes, 2011; Perry et al., 2011). Fully evaluating the effects of this was out of the scope of the present study. We argue that these effects are not expected to underlie the main results of the cross-method comparisons reported here. For example, the identical MEG-fMRI co-registration method and forward model specification was used for the beamformer and MSP approaches within the SPM8 software package, and a near-identical method was also used for MNE. The same anatomical surfaces and digitizer points were used for all reconstructions.

## Conclusions

MEG source reconstructions with prior assumptions of many independent, distributed sources of small amplitude (in connection with individual anatomical mesh data), or with prior assumptions of a spatial filtering (beamformer) approach, seem well matched to localize the irregularly patterned, unilateral responses of retinotopic subregions of the early visual areas. On the other hand, the sparse priors of the MSP method may be better matched for large, cluster-like source distributions that are bilateral, such as responses to face stimuli in the fusiform gyrus (Henson et al., 2009). Sources in early visual areas are more accurately localized according to angular retinotopy rather than eccentricity. Stimuli should be confined to a visual field quadrant (i.e., not bilateral). Further work is necessary to tease out the quantitative contributions of different prior assumptions and source space constructions. However, researchers aiming to localize brain activity arising from the early visual regions should take spatial extent into account when designing the stimulus and should carefully match the analysis method and software package used to the expected distribution of the signal.

### Conflict of interest statement

The authors declare that the research was conducted in the absence of any commercial or financial relationships that could be construed as a potential conflict of interest.
